# Antimicrobial activity of D-amino acid in combination with photo-sonoactivated hypericin nanoparticles against *Acinetobacter baumannii*

**DOI:** 10.1186/s12866-023-02758-4

**Published:** 2023-01-19

**Authors:** Maryam Pourhajibagher, Nava Hosseini, Abbas Bahador

**Affiliations:** 1 Dental Research Center, Dentistry Research Institute, Tehran University of Medical Sciences, Tehran, Iran; 2 Institut de Biologie Intégrative et des Systèmes (IBIS), Pavillon Charles-Eugène-Marchand, Université Laval, Quebec City, QC G1V 0A6 Canada; 3 Département de Biochimie, de Microbiologie et de Bio-Informatique, Faculté des Sciences et de Génie, Université Laval, Quebec City, QC G1V 0A6 Canada; 4 Centre de Recherche de l’Institut Universitaire de Cardiologie et de Pneumologie de Québec (IUCPQ), Quebec City, QC G1V 4G5 Canada; 5 Fellowship in Clinical Laboratory Sciences, BioHealth Lab, Tehran, Iran; 6 Department of Microbiology, Tehran University of Medical Sciences, Tehran, Iran

**Keywords:** *Acinetobacter baumannii*, Antimicrobial photodynamic therapy, Antimicrobial sonodynamic therapy, Biofilms, Drug resistance, Nosocomial infection

## Abstract

**Background:**

The emergence of multidrug-resistant *Acinetobacter baumannii* strains is increasing worldwide. To overcome these life-threatening infections, the development of new treatment approaches is critical. For this purpose, this study was conducted to determine the antimicrobial photo-sonodynamic therapy (aPSDT) using hypericin nanoparticles (HypNP) in combination with D-Tryptophan (D-Trp) against *A. baumannii*.

**Materials and methods:**

HypNP was synthesized and characterized, followed by the determination of the fractional inhibitory concentration (FIC) index of HypNP and D-Trp by checkerboard assay. Next, the antimicrobial and anti-biofilm potential of HypNP@D-Trp-mediated aPSDT against *A. baumannii* was evaluated. Finally, the anti-virulence activity of aPSDT using HypNP@D-Trp was accessed following the characterization of HypNP@D-Trp interaction with AbaI using in silico virtual screening and molecular docking.

**Results:**

A synergistic activity in the combination of HypNP and D-Trp against *A. baumannii* was observed with a FIC index value of 0.5. There was a 5.10 log_10_ CFU/mL reduction in the cell viability of *A. baumannii* when the bacterial cells were treated with 1/2 × MIC of HypNP@D-Trp and subsequently exposed to ultrasound waves and blue light (*P* < 0.05). Moreover, a significant biofilm degradation effect on biofilm-associated cells of *A. baumannii* was observed after treatment with aPSDT using 2 × MIC of HypNP@D-Trp in comparison with the control groups (*P* < 0.05). According to the molecular docking analysis of the protein-ligand complex, Hyp with a high affinity for AbaI showed a binding affinity of − 9.41 kcal/mol. Also, the expression level of *abaI* gene was significantly downregulated by 10.32-fold in *A. baumannii* treated with aPSDT as comprised with the control group (*P* < 0.05).

**Conclusions:**

It can be concluded that HypNP@D-Trp-mediated aPSDT can be considered a promising strategy to overcome the infections caused by *A. baumannii* by reducing the growth of bacterial biofilm and decreasing the expression of *abaI* as a gene involved in *A. baumannii* biofilm formation.

## Introduction

Multidrug-resistant (MDR) and extensively drug-resistant (XDR) bacteria have emerged as formidable nosocomial pathogens, particularly among patients in intensive care units (ICU). In developing countries, clinicians face serious challenges in treating critically ill patients with MDR and XDR bacterial infections. In antibiotic-resistant Gram-negative bacteria, beta-lactamases are considered the primary antibiotic resistance mechanisms [1], especially Class D OXA-type beta-lactamases are the most prevalent carbapenemases in *Acinetobacter baumannii* [2]. However, in *A. baumannii* like *Klebsiella pneumoniae*, extended-spectrum β-lactamases (ESBLs) and AmpC β-lactamases (MOX, ACT, and FOX genes) are clinically significant because they may confer high-level resistance to a wide variety of β-lactam antibiotics [1, 3]. However, not only is resistance to antimicrobials widespread in developing countries, but also the newer effective antimicrobial agents are costly and less accessible. Thus, to address the need for effective and affordable antimicrobials to control outbreaks and treat patients with MDR and XDR bacterial infections, in recent years, ongoing research has focused on the investigation and development of nano-scale objects as efficient antimicrobial agents. However, concerns about the synthesis of these agents such as the use of toxic solvents and precursor chemicals, and the generation of toxic byproducts have led to a green synthesis approach including the use of lichens for the biosynthesis of silver (Ag) nanoparticles (NPs) [4] and usage of herbal derivative or extracts from medicinal plant species based on micro and nanoformulations including embelin and Hypericin which are found in red berry fruits of the *Embelia ribes* species [5] and St. John’s Wort (*Hypericum* species) [6], respectively.

*A. baumannii* is one of the most difficult-to-treat pathogens. It is a causative agent of a wide range of nosocomial infections associated with multidrug resistance and high rates of morbidity and mortality [7, 8]. Numerous factors such as cell density sensing, protein glycosylation systems, auto-inducer synthase, concentrations of free cations, poly-β-(1–6)-N-acetyl glucosamine extracellular polysaccharide, and biofilm-associated proteins are involved in the pathogenesis of *A. baumannii* [9, 10]. Quorum sensing (QS) is a process of cell-to-cell communication in that bacteria coordinate their group behavior by sensing their cell density [11]. The *A. baumannii* QS system consists of AbaIR, AbaI, and its regulator AbaR, which mainly focuses on biofilm formation, motility, and antibiotic resistance [12]. Moreover, *A. baumannii* strains have evolved both physiological and genetic mechanisms to survive in diverse environments and tolerate extremely different conditions [13]. Therefore, there is a critical need to develop new treatment approaches against these life-threatening infections.

Antimicrobial peptides (AMPs) are effector molecules of innate immunity due to their pleiotropic functions in microbial killing, inflammation, angiogenesis, and wound healing, and promising alternatives to conventional antibiotics [14]. AMPs include many different classes such as those that are rich in particular amino acids. Several studies suggest that certain amino acids may negatively affect bacterial metabolism. Solutes such as these D-amino acids not only are similar in structure to compatible solutes but also display an opposite physiological effect on bacterial growth rate [15–18]. One of the important subsets is peptides rich in Tryptophan (Trp) which has broad and potent antimicrobial activity [17–19]. As proven, Trp residues have a strong membrane-disruptive activity and this property enables Trp-containing antimicrobial agents to interact with the surface of microbial cell membranes, possibly improving antimicrobial properties [19].

In order to enhance the effectiveness of AMPs against microorganisms, various approaches such as antimicrobial photodynamic therapy (aPDT) and antimicrobial sonodynamic therapy (aSDT) can be used [20, 21]. Both aPDT and aSDT are techniques that have been shown to be effective in vivo and in vitro against microbial pathogens [22–26]. aPDT involves the application of a photosensitizing agent, low-level light energy at a specific wavelength, and the presence of oxygen [27]. aSDT uses the sensitization of the target site with a non-toxic sonosensitizer, relatively low-intensity ultrasound waves, and molecular oxygen which produce the microbubbles through the acoustic cavitation process during the interactions between the ultrasound wave and target cells [28]. According to studies, some nanomaterials such as hypericin nanoparticles (HypNPs) are photo-sonosensitizers and can excite after exposure to visible light and/or ultrasound waves [29–33]. During this process, called antimicrobial photo-sonodynamic therapy (aPSDT), the excited photo-sonosensitizer generates reactive oxygen species (ROS), resulting kill cells by damaging biomolecules such as proteins, nucleic acids, and lipids [34].

The available literature indicates that there was no evidence of the application of aPSDT against *A. baumannii.* In the current study, the combination of HypNP with D-Trp (HypNP@D-Trp) was used to further improve the antimicrobial activities of aPSDT against *A. baumannii*. The antimicrobial and anti-biofilm activities as well as the anti-virulence potency of aPSDT using HypNP@D-Trp were accessed following the characterization of HypNP@D-Trp interaction with AbaI using in silico computational models. It was hypothesized that HypNP@D-Trp improves the antimicrobial and anti-biofilm effects of aPSDT against *A. baumannii*. Moreover, HypNP@D-Trp could be interacted with AbaI with high affinity and subsequently reduced the gene expression level of *abaI*.

The novelty of the current study lies in introducing an aPSDT approach based on a new formulation of HypNP with D-Trp (HypNP@D-Trp), as a photo-sonosensitizer was accessed following the characterization of HypNP@D-Trp interaction with AbaI using in silico computational models to reduce the population and virulence of *A. baumannii* as one of the most resistant bacteria. The results of the current study add to the literature in highlighting the importance and efficacy of the HypNP@D-Trp mediated aPSDT, to the reduction of the *A. baumannii* population and its virulence which results in better management of local *A. baumannii* infections such as nosocomial wound infections. So, this study aims to investigate the anti-virulence, and antimicrobial activities against *A. baumannii*, of HypNP@D-Trp, mediated aPSDT following the characterization of HypNP@D-Trp interaction with AbaI using in silico computational models. The null hypothesis is that HypNP@D-Trp mediated aPSDT has no antimicrobial and anti-virulence effects against *A. baumannii*.

## Material and methods

### Preparation and characterization of HypNP

The synthesis of HypNP was performed according to a method previously described, with slight modifications [35]. Briefly, 4 mg/mL Hyp (purity ≥98%, Sigma-Aldrich, Germany) prepared in 95% ethanol was dissolved in 2 mL tetrahydrofuran (THF; Sigma-Aldrich, Germany) under constant magnetic agitation. After stirring for a definite time, the ethanol and THF were evaporated and the HypNP solution was obtained by filtration through a 0.22 μm syringe filter.

A transmission electron microscopy (TEM) image of HypNP was taken by a transmission electron microscope (TEM; Zeiss EM10C) operating at 80 kV in imaging mode. Also, the hydrodynamic size and size distribution were acquired by dynamic light scattering (DLS, Malvern Instruments Ltd., UK).

### D-amino acid

D-Trp was purchased from Sigma–Aldrich (Steinheim, Germany). The stock solution was prepared in water followed by filter sterilization using a 0.22 μm syringe filter.

### Bacterial strain and growth conditions

*A. baumannii* ATCC 19606 used in this study was obtained from the National Microbial Bank of Iran, Pasteur Institute (Tehran, Iran). *A. baumannii* was grown in brain heart infusion (BHI) broth (Merck, Germany) under aerobic conditions at 37 °C in a shaking incubator to reach a mid-logarithmic phase corresponding to an OD 600 nm of 0.08–0.13 which approximated to 1.5 × 10^8^ colony forming units (CFU)/mL).

### Checkerboard assay

The combination effect of HypNP with D-Trp was determined by calculating the fractional inhibitory concentration index (FICI) as previously reported [36]. The concentrations of D-Trp for minimal inhibitory concentration (MIC) determination were 6.2, 12.5, 25, 50, and 100 mM, and HypNP: 8, 16, 32, 64, AND 128 μg/mL. The FICI was determined as follows:

$$\textrm{FICI}=\frac{{\textrm{MIC}}_{\textrm{A}}^{\textrm{combination}}}{{\textrm{MIC}}_{\textrm{A}}^{\textrm{alone}}}+\frac{{\textrm{MIC}}_{\textrm{B}}^{\textrm{combination}}}{{\textrm{MIC}}_{\textrm{B}}^{\textrm{alone}}}$$

The antimicrobial combination was defined to be synergistic when the FICI was ≤ 0.5; additive or indifferent when > 0.5 to 4; and antagonistic when FICI > 4.

### Light source

Blue light at the wavelength of 450 ± 10 nm, an optical output power of 100 mW, and an energy density of 25.56 J/cm^2^ for an exposure time of 60 s was used as the light source. According to the previous study [37], the probe of the blue light was fixed on a laboratory stand at a 1-mm distance above each well’s surface. To prevent beam reflection from the tabletop during irradiation, a black sheet was placed under the microtiter plate.

### Ultrasound irradiation system

Herein, an ultrasonic apparatus set had a transducer with a diameter of 3.5 cm and an ultrasound frequency of 30 KHz. The microtiter plate was vertically placed at a distance of 5 cm from the transducer.

### The effect of treatment groups on the growth rate of *A. baumannii*

The growth rate of *A. baumannii* was determined as previously reported [38]. Briefly, 100 μL of *A. baumannii* suspension at the concentration of 1.5 × 10^6^ CFU/mL were poured into the wells of a 96-well microtiter plate. Next, the bacterial cells were treated with the experimental groups as follows:

A. HypNP: 100 μL of HypNP at 1/2 × MIC was added to the bacterial cells and the microtiter plate was incubated in the dark at room temperature for 5 min.
B. D-Trp: 100 μL of D-Trp at 1/2 × MIC was added to the bacterial cells and the microtiter plate was incubated in the dark at room temperature for 5 min.
C. HypNP@D-Trp: 100 μL of HypNP@D-Trp at 1/2 × MIC was added to the bacterial cells and the microtiter plate was incubated for 5 min.
D. Blue light: 100 μL of BHI broth was added to the bacterial cells and *A. baumannii* was exposed to the blue light at the wavelength of 450 ± 10 nm for 60 s.
E. Ultrasound waves: 100 μL of BHI broth was added to the bacterial cells and *A. baumannii* was exposed to the ultrasound waves with the ultrasound frequency of 1 MHz and pulse repetition frequency of 100 Hz for 60 s.
F. aPDT: *A. baumannii* suspension was separately treated by HypNP, D-Trp, and HypNP@D-Trp similar to groups A-C, respectively. Then the cells were exposed to blue light similar to group D.
G. aSDT: *A. baumannii* suspension was separately treated by HypNP, D-Trp, and HypNP@D-Trp similar to groups A-C, respectively. Then the cells were exposed to the ultrasound wave similar to group E.
H. aPSDT: *A. baumannii* suspension was separately treated by HypNP, D-Trp, and HypNP@D-Trp similar to groups A-C, respectively. Then the cells were exposed to the blue light similar to group D and subsequently ultrasound wave similar to group E.
I. Positive control: 100 μL of silver sulfadiazine (SSD) (1% w/w as the standard treatment) was added to the bacterial cells and incubated at room temperature for 5 min.
J. Negative control: 100 μL of normal saline was added to the bacterial cells and incubated for 5 min at room temperature.

After each treatment, the bacterial cells were serially 10-fold diluted with medium and the log_10_ CFU/mL values were calculated after the plates were incubated at 37 °C for 24 h.

### The effect of treatment groups on *A. baumannii* biofilms

The method of biofilm formation on the bottom of a 96-well flat-bottom microtiter plate was conducted by a previously described method [39]. Briefly, 200 μL of *A. baumannii* at the concentration of 1.0 × 10^8^ CFU/mL was added to each well of the microtiter plate. The plate was incubated for 24 h at 37 °C for biofilm growth. After that, the biofilms formed on the microtiter plate were washed with phosphate-buffered saline (PBS) to remove planktonic cells, and adherent bacteria were treated with the experimental groups (A-J) as mentioned above, except that in all groups, 2 × MIC of HypNP, D-Trp, and HypNP@D-Trp were used. The treated bacteria were then fixed with 95% ethanol and stained with 0.1% crystal violet for 10 and 15 min, respectively. To measure biofilm degradation, crystal violet was solubilized by adding 100 μL acetic acid (33%). The absorbance of the solubilized dye was measured at 570 nm and the percentage of biofilm degradation was determined by the following equation:

$$\textrm{Biofilm}\ \textrm{degradation}=\frac{\textrm{OD}\ \textrm{of}\ \textrm{untreated}\ A. baumanii-\textrm{OD}\ \textrm{of}\ \textrm{sample}\ }{\textrm{OD}\ \textrm{of}\ \textrm{untreated}\ A. baumanii}\times 100$$

### Construction and validation of the homology model

The amino acid sequences of AbaI were gained from National Center for Biological Information (http://www.ncbi.nlm.nih.gov/). The selected sequence was subjected to the Protein-Basic Local Alignment Search Tool (BLASTP; https://www.ncbi.nlm.nih.gov/blast/) to retrieve similar sequences to the query sequence. The three-dimensional structure of AbaI was retrieved from the Protein Data Bank with PDB ID 3P2F (https://www.rcsb.org/structure/3P2F). The quality of the model structure was confirmed using ERRAT and Verify_3D programs and the authenticity of the model was determined by Ramachandran scores (https://www.ebi.ac.uk/thornton-srv/software/PROCHECK/). The SWISS-MODEL structure assessment online server was used for validation of the model coordinates (https://swissmodel.expasy.org/).

### Protein-protein interactions (PPIs) network analysis

STRING (http://string-db.org) was used to predict PPIs including direct (physical) and indirect (functional) associations and rank their significance or validity. The query sequence was analyzed to determine the family of proteins using the motif finder server (http://www.genome.jp/tools/motif/). Moreover, the Protter server (http://wlab.ethz.ch/protter/) was then used to assess the interactive protein feature visualization.

### Ligand’s retrieval

In the current study, Hyp was used as the ligand and its three-dimensional structure was obtained from PubChem (www.pubchem.com) in .sdf format.

### Molecular docking analysis

The simulation of molecular docking was performed on the SwissDock server (http://www.swissdock.ch/docking) to examine the potential binding mode of ligand to AbaI. The best interaction binding energy (kcal/mol) was selected for evaluation and analysis according to the potential intermolecular interactions between protein and ligand.

### RNA isolation, reverse transcription, and quantitative real-time PCR

Quantitative real-time polymerase chain reaction (qRT-PCR) was used to determine the changes in *abaI* gene expression of *A. baumannii*. Immediately after treatment of *A. baumannii* with sub-MIC doses of HypNP, D-Trp, and HypNP@D-Trp, followed by exposure to blue light and ultrasound wave as mentioned in the section “Effect of treatment groups on the growth rate of *A. baumannii*”, the total RNA was extracted using the super RNA extraction kit (AnaCell, Iran) following the manufacturer’s instructions. Total RNA (150 ng) was then used to synthesize cDNA using the cDNA synthesis kit (AnaCell, Iran) as per the manufacturer’s recommendations.

The specific primers related to *abaI* and *16S rRNA* genes listed in Table 1 were designed using Primer3Plus software version 4.0 (https://www.bioinformatics.nl/cgi-bin/primer3plus/primer3plus.cgi). The *16S rRNA* gene was used as an internal control for the normalization of gene expression levels. The real-time PCR reaction was conducted in the ABI Thermocycler System (ABI Step One™, USA) with the following cycle profile: One cycle at 95 °C for 5 min followed by 40 cycles at 95 °C for 20 s, annealing at 55 °C for 10 s, and extension at 72 °C for 10 s. Eventually, the relative gene expression levels were calculated using Livak and Schmittgen (2^−ΔΔCT^) method [40].

**Table 1:** Primer sequences used in this study

Genes		Sequences (5́ − 3́)^**a**^	Amplicon Size (bp)
***abaI***	F	TGGGTTGGGAGTTGAACTGT	137
	R	GGGTTGTGTGGTGGGTAGTA	
***16S rRNA***	F	AAAGTTGGTATTCGCAACGG	117
	R	ACCTTTAACCCGCTTTTGCT	

*Abbreviations*: *F* Forward primer, *R* Reverse primer; and *bp* Base pair

^a^Nucleotides

### Statistical analysis

All experiments were performed in triplicates and the values are expressed as mean ± standard deviation. The data were statistically analyzed by one-way ANOVA with Tukey post hoc test by the SPSS software, version 23.0. *P* values of less than 0.05 were considered statistically significant.

## Results

### Confirmation of HypNP synthesis

**Fig. 1:**
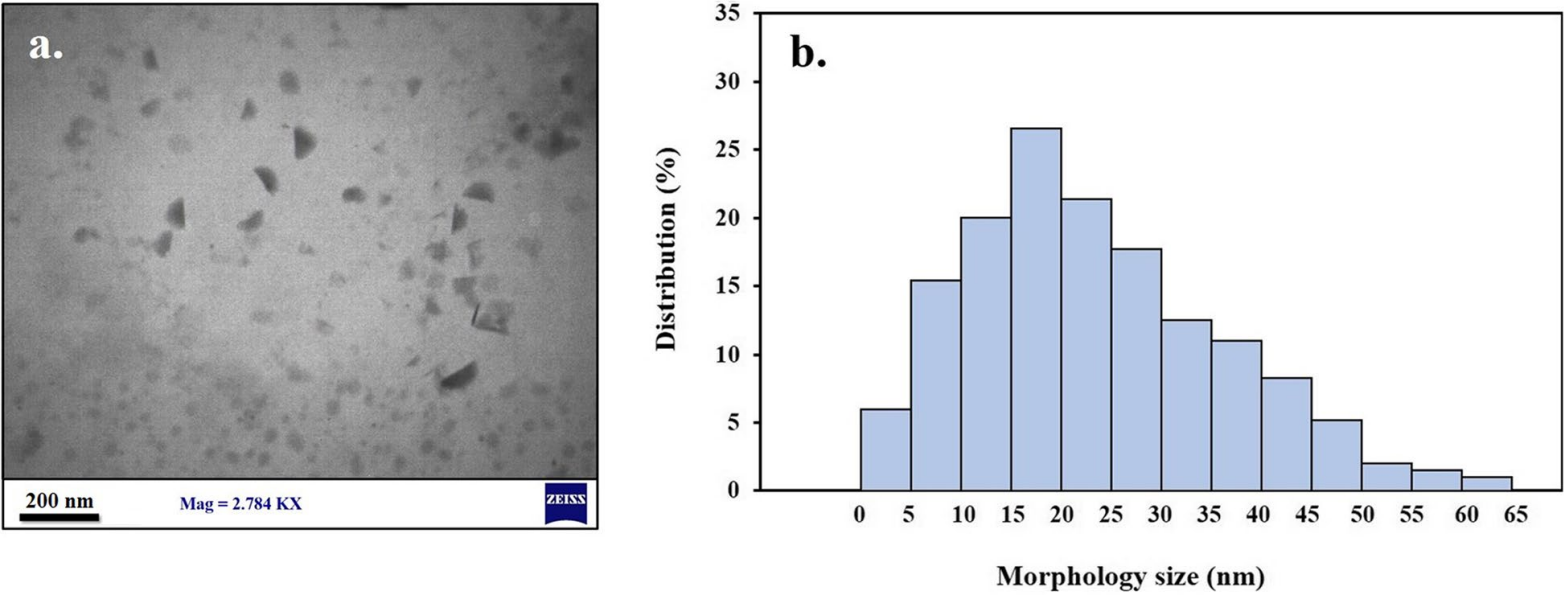
Characterization of synthesized hypericin nanoparticle (HypNP): a) Transmission electron microscope (TEM) image (Scale bar = 200 nm), b) The size distribution profile of HypNP

The TEM image illustrated that the morphology and size of HypNPs are quasi-spherical in shape with diverse sizes (Fig. 1a). As shown in Fig. 1b, HypNP had an average size of 5–65 nm with a mean size of 24.6 ± 2.8 nm.

### Checkerboard assay

The checkerboard assay was conducted using D-Trp and HypNP against *A. baumannii.* Results showed the MIC value for D-Trp and HypNP with MIC ≥50 mM, and MIC ≥64 μg/mL, respectively. FICI was calculated using the concentration combinations with the highest combination effects, that is 12.5 mM of D-Trp or 16 μg/mL of HypNP. According to the results, synergy in the checkerboard method was seen in D-Trp and HypNP combinations against *A. baumannii*.

### Cell viability of *A. baumannii* in the planktonic state

**Fig. 2:**
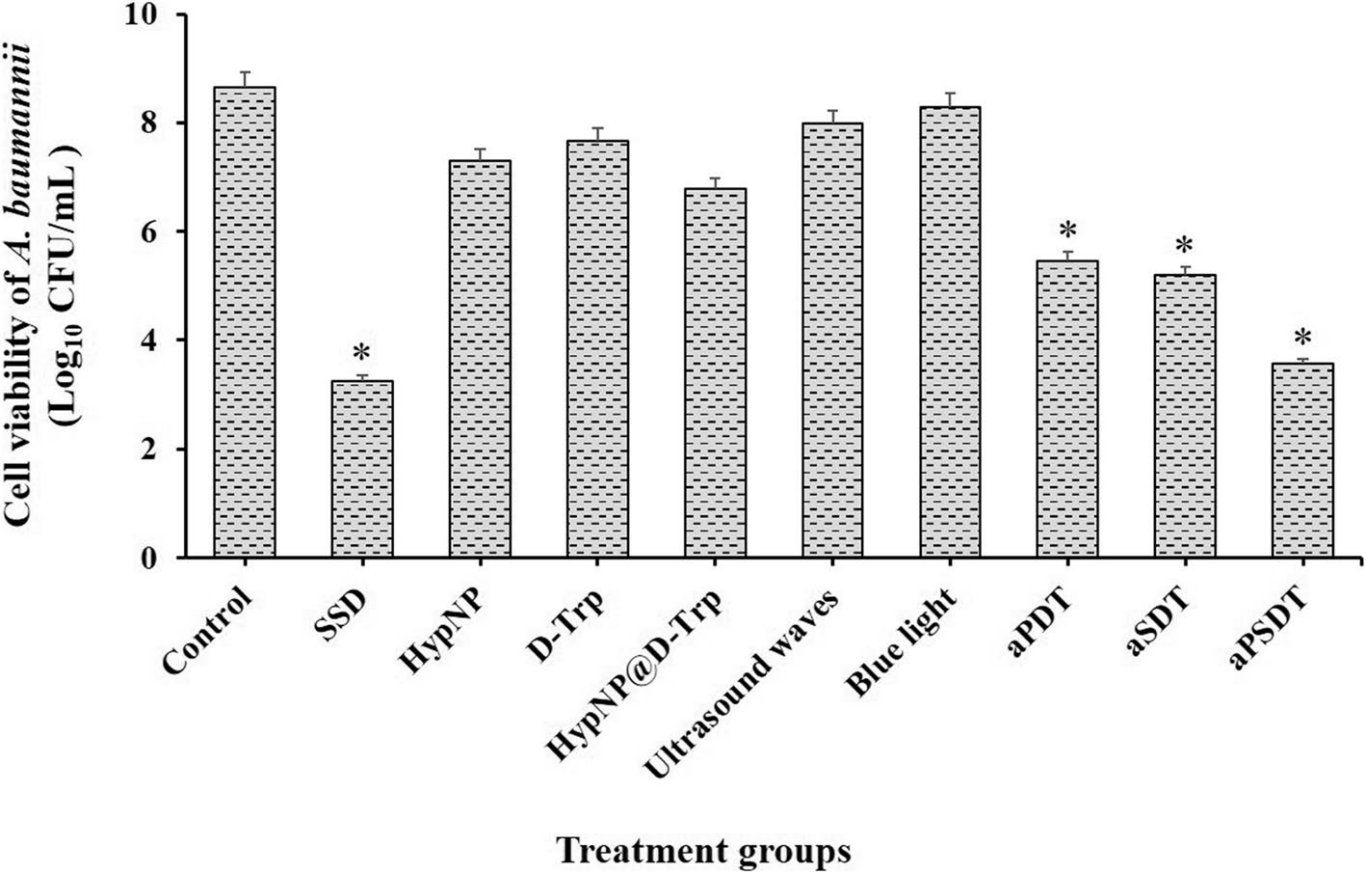
Effects of different treatment groups on the cell viability of *Acinetobacter baumannii.* *Significantly different from the control group (no treatment), *P* < 0.05

The results of planktonic cultures showed that aPDT, aSDT, and aPSDT using 1/2 × MIC of HypNP@D-Trp significantly reduced the number of viable cells of *A. baumannii* compared to the control group (non-treated group; *P* < 0.05; Fig. 2). As shown in Fig. 2, there was no significant difference between SSD with aPSDT groups (*P* > 0.05). HypNP, D-Trp, HypNP@D-Trp, ultrasound waves, and blue light alone were ineffective against *A. baumannii* strains in planktonic form and only caused less than 2 log_10_ CFU/mL reduction in the number of viable bacteria (*P* > 0.05).

### Cell viability of *A. baumannii* in the biofilm state

**Fig. 3:**
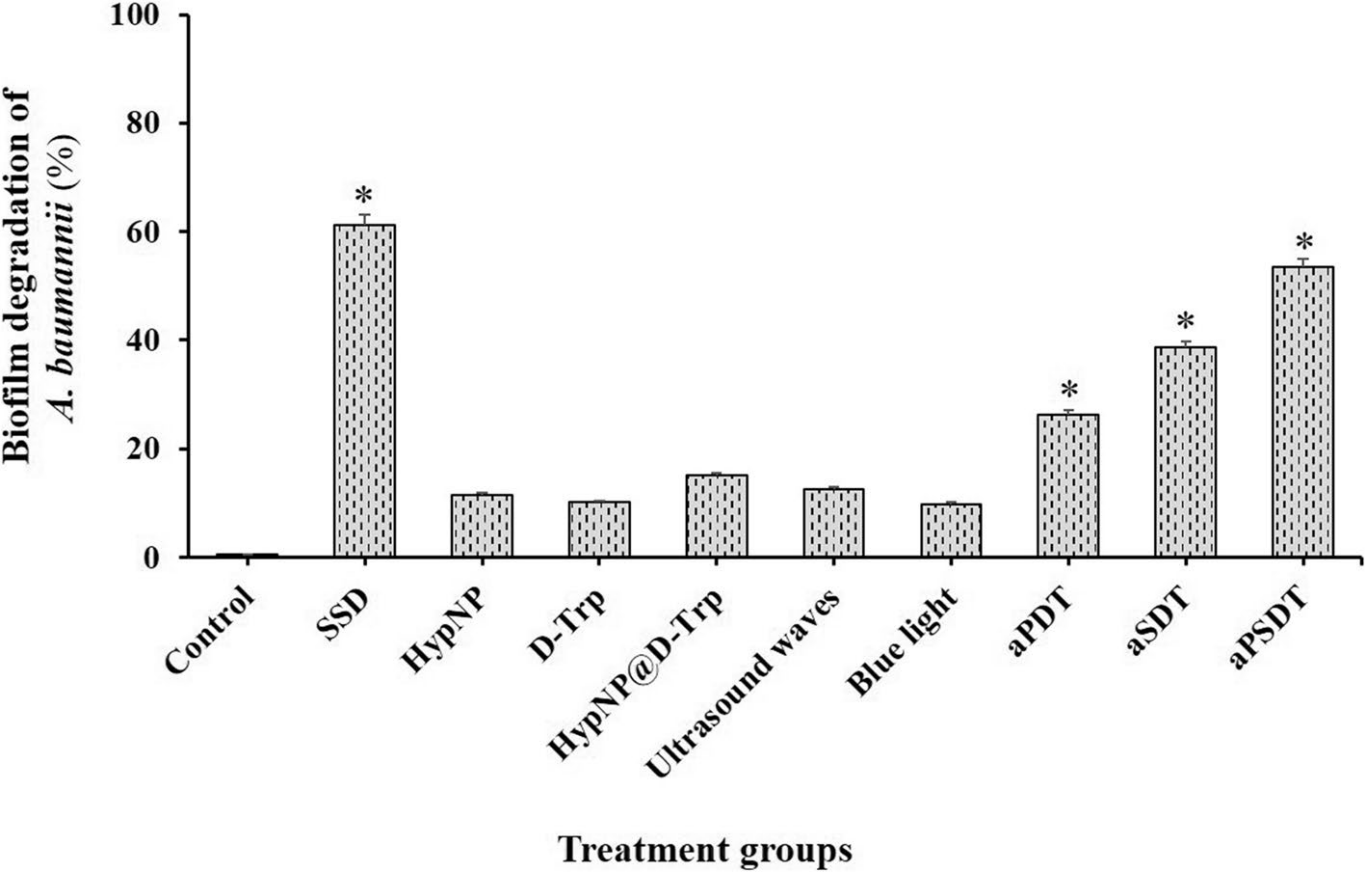
Effects of different treatment groups on the biofilm of *Acinetobacter baumannii.* *Significantly different from the control group (no treatment), *P* < 0.05

According to the results of Figs. 3, 2×MIC of HypNP, D-Trp, and HypNP@D-Trp did not cause a biofilm degradation effect on biofilm-associated cells of *A. baumannii* (*P* > 0.05). However, the significant destructive effect on biofilm of this strain was observed after treatment with aPDT, aSDT, and aPSDT as compared with the control group (*P* < 0.05). Also, ultrasound waves and blue light alone did not show a significant effect on the viability of *A. baumannii* biofilm (*P* > 0.05).

### Virtual screening

Basic information obtained from AbaI showed that it has 188 amino acids with a molecular weight of 20,593.63 Da. The amino acid compositions of AbaI are Alanine (Ala) 8.2%; Arginine (Arg) 6.0%; Asparagine (Asn) 4.9%; Aspartic acid (Asp) 3.3%; Cysteine (Cys) 2.2%, Glutamine (Gln) 4.9%; Glutamic acid (Glu) 6.5%; Glycine (Gly) 6.0%; Histidine (His) 0.5%; Isoleucine (Ile) 8.2%; Leucine (Leu) 8.7%; Lysine (Lys) 2.7%; Methionine (Met) 2.7%; Phenylalanine (Phe) 6.0%; Proline (Pro) 7.1%; Serine (Ser) 8.7%; Threonine (Thr) 3.8%; Tryptophan (Trp) 1.1%; Tyrosine (Tyr) 3.8%; and Valine (Val) 4.9%. In silico computational analysis showed that the number of positively charged residues (Arg + Lys) and negatively charged residues (Asp + Glu) of AbaI are 16 and 18, respectively. The extinction coefficient of AbaI at 280 nm in water was found to be 21,680 M^− 1^ cm^− 1^. Also, the theoretical isoelectric point (pI) and the grand average of hydropathicity (GRAVY) of AbaI were computed to 5.47 and − 0.047, respectively.

### Model evaluation

**Fig. 4:**
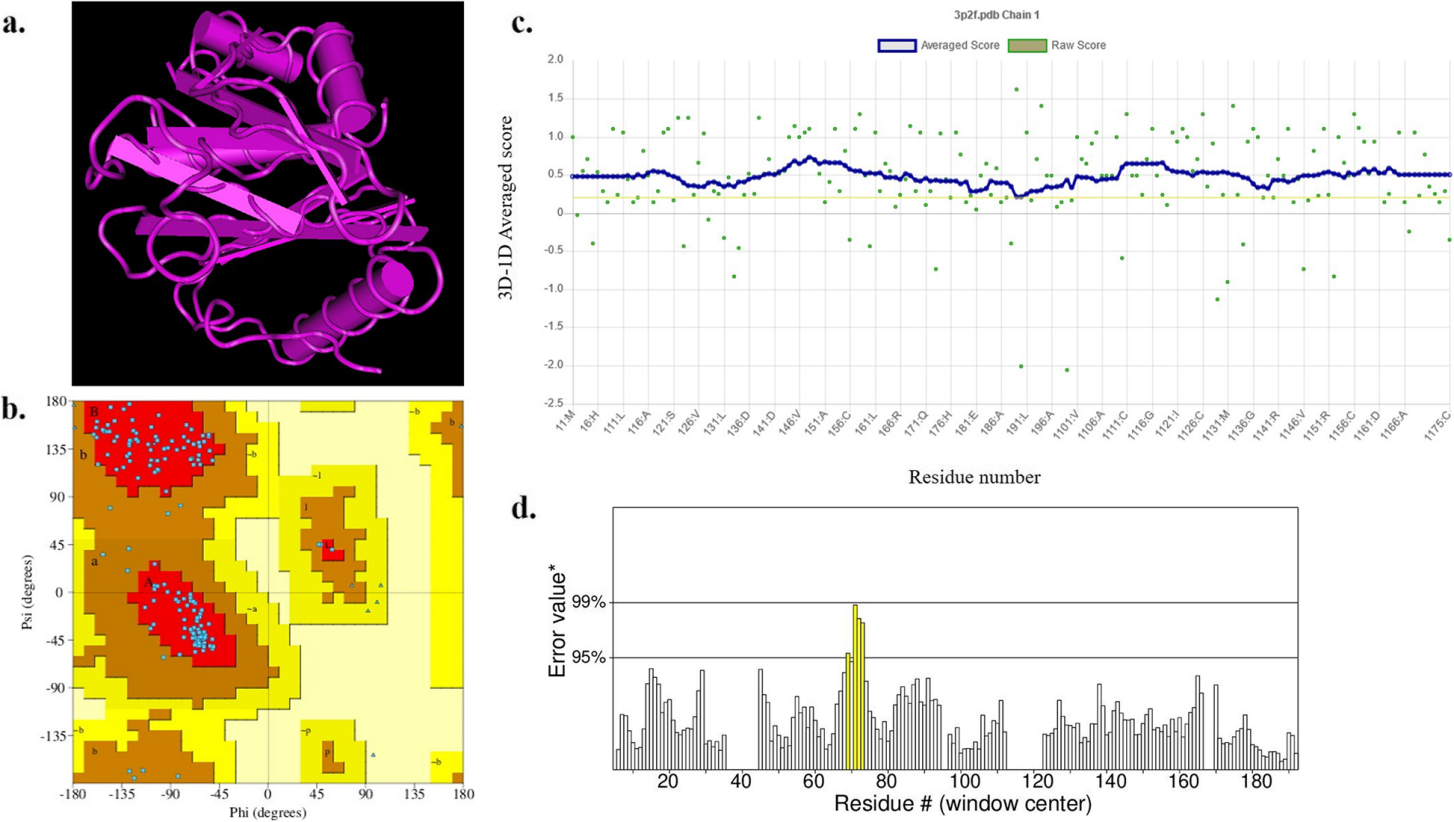
Confirmation of quality of AbaI structure by a. The three-dimensional modeling of 3P2F; b. The Ramachandran plot of 3P2F; c. Verify_3D; and d. ERRAT

The sequence of AbaI was blasted in PDB to identify proteins having sequences similar to AbaI. The results showed that AbaI is similar to the protein structure with accession number 3P2F. The three-dimensional modeling of 3P2F is shown in Fig. 4a. The resolution of the retrieved structure was 2.30 Å. The modeled tertiary structure was analyzed with PROCHECK, which predicted that 90.7% of 3P2F residues are in favored regions and 9.3% in additional allowed regions (Fig. 4b). VERIFY-3D result showed that 100% residues had averaged 3D-1D score > = 0.2 and at least 80% of the amino acids had scored > = 0.2 in the 3D/1D profile (Fig. 4c). The overall quality factor of the model predicted by ERRAT was 97.546, which implicated that any bonded atomic interactions of the generated model were not within the normal range (Fig. 4d). Hence, results obtained from PROCHECK, ERRAT, and VERIFY-3D indicate that the modeled structure of AbaI is reasonable and reliable as a target for HypNP in HypNP@D-Trp.

### PPIs network analysis

**Fig. 5:**
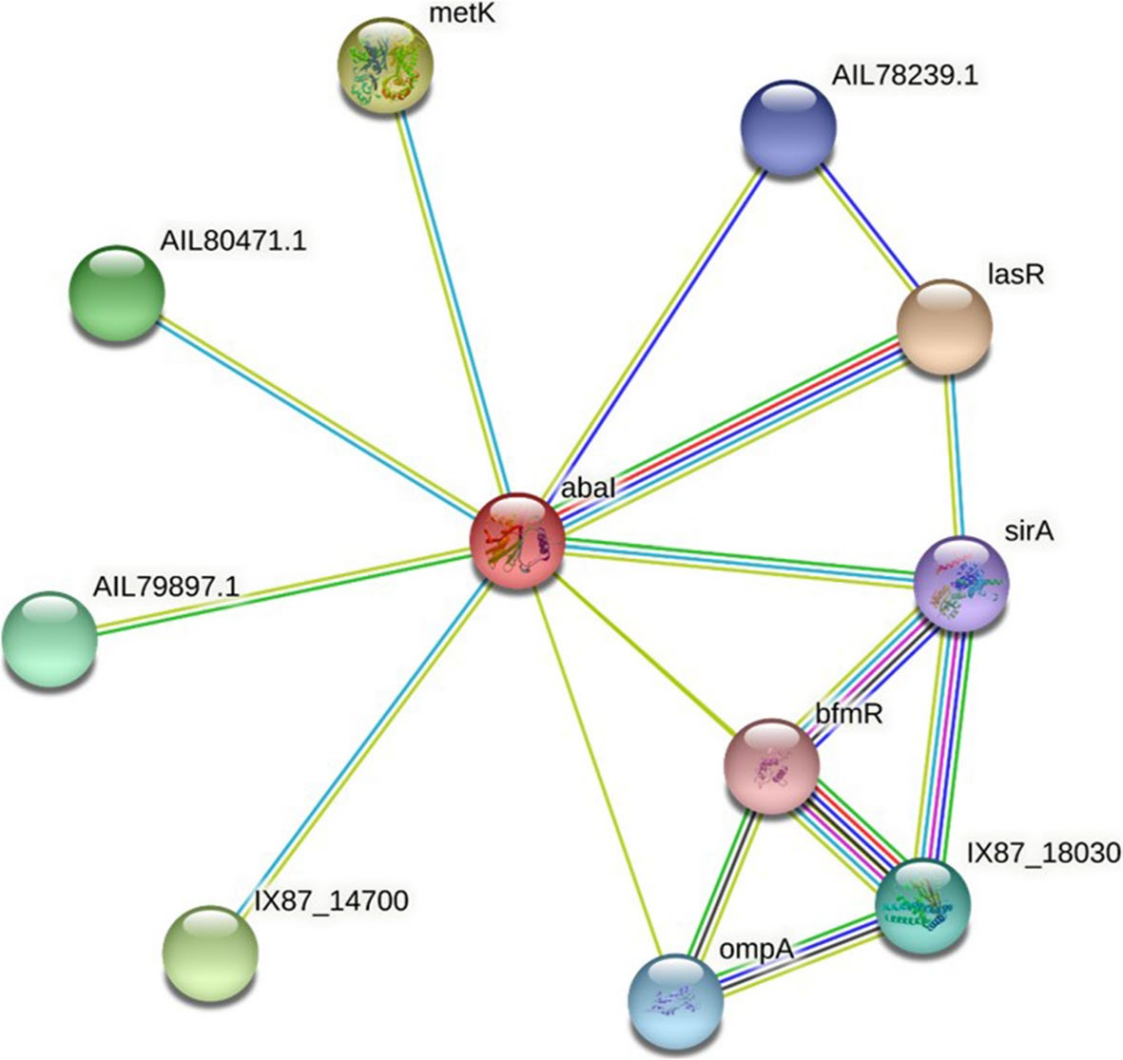
Protein-protein interactions network analysis of AbaI

The PPIs network has displayed proteins and their length and type of relationship with AbaI protein (Fig. 5). The number of nodes and edges were 11 and 17, respectively. According to the in silico findings, the average node degree was 3.09 and the PPI enrichment *P*-value and the average local clustering coefficient were 0.0396 and 0.832, respectively. As shown in Fig. 5, AbaI has several predicted functional partners, such as lasR (Luxr family transcriptional regulator; with a score of 0.990), metK (S-adenosylmethionine synthetase; with a score of 0.931), IX87_14700 (5́-nucleosidase; with a score of 0.928), AIL80471.1 (5́-methylthioadenosine nucleosidase; with a score of 0.928), AIL79897.1 (Hypothetical protein; with a score of 0.657), IX87_18030 (Two-component sensor histidine kinase; with a score of 0.612), ompA (annotation not available; with a score of 0.603), AIL78239.1 (Lactonase; with a score of 0.595), and SirA (BarA-associated response regulator UvrY; with a score of 0.594), whatever the protein score is closer to 1 is an indication that this protein is near to AbaI.

### Molecular docking analysis

**Fig. 6:**
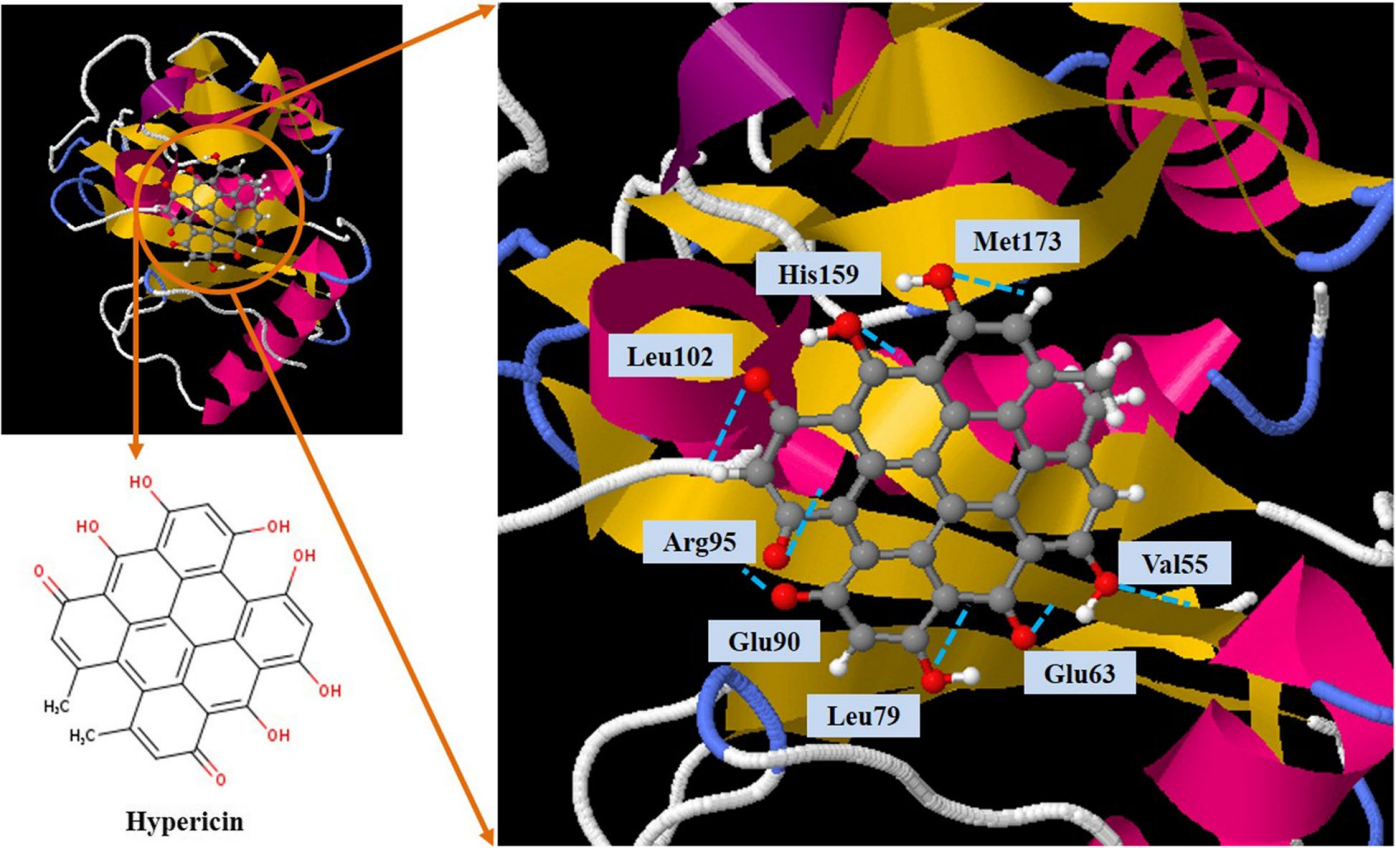
Representation of docked ligand-protein complex; Interaction of Hypericin with amino acid residues of AbaI

In this study, in silico molecular docking was performed to estimate the binding energy and demonstrate the protein-ligand interaction. According to the data, Hyp showed the best interaction with the binding affinity of − 9.41 kcal/mol. It was found to interact with AbaI at active sites Val55, Glu63, Leu79, Glu90, Arg95, Leu102, His159, and Met173 (Fig. 6).

### Anti-virulence effect of treatment groups against *A. baumannii*

**Fig. 7:**
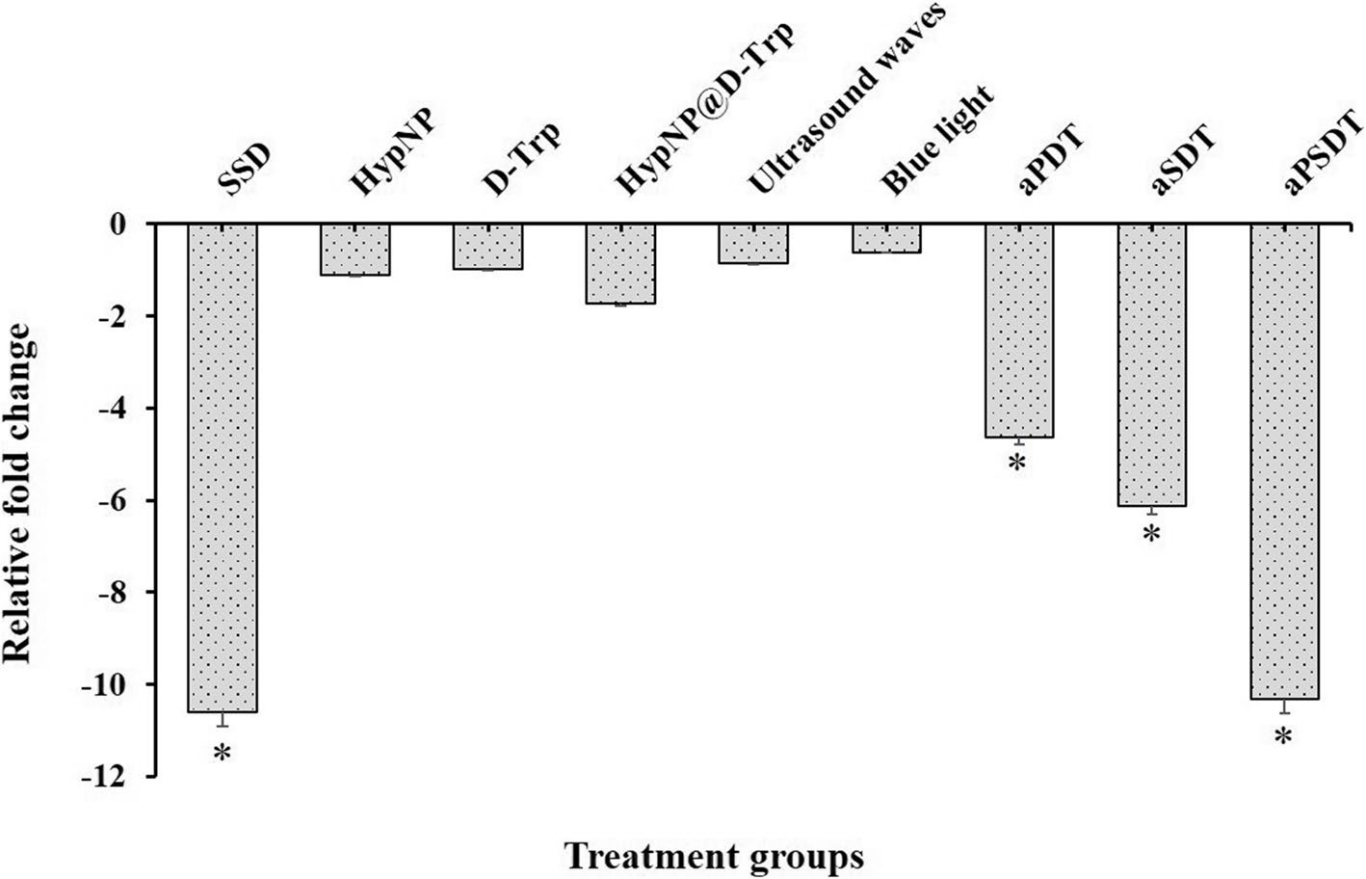
Effects of different treatment groups on the expression of gene involved in biofilm formation of *Acinetobacter baumannii.* *Significantly different from the control group (no treatment), *P* < 0.05

As shown in Fig. 7, aPDT, aSDT, and aPSDT using 1/2 × MIC of HypNP@D-Trp significantly downregulated the expression level of *abaI* to 4.64, 6.12, and 10.32-folds, respectively (*P* < 0.05). Following treatment with ultrasound waves and blue light groups, the expression of *abaI* was stable and not significant (*P* > 0.05) but was slightly higher than in the HypNP, D-Trp, and HypNP@D-Trp-treated groups. Although *abaI* was found to be downregulated in response to HypNP, D-Trp, and HypNP@D-Trp, this reduction is non-significant (*P* > 0.05). According to the findings, no significant difference was observed in the gene expression level of *abaI* between SSD with aPSDT groups (*P* > 0.05).

## Discussion

The emergence of drug-resistant *A. baumannii* strains seriously challenges the treatment of these infections [41]. aPDT and aSDT have shown potential to be used as adjuvant therapeutic against microbial infections [22–26]; however, their success is dependent on the efficiency of the photosensitizer and sonosensitizer employed.

In the current study, HypNP@D-Trp was used. Hyp (4,5,7,4′,5′,7′-hexahydroxy-2,2′-dimethyl-naphthodianthrone) is an anthraquinone derivative and one of the main active compounds in St. John’s wort (*Hypericum perforatum* L.) [42]. There are several studies assessing the application of Hyp for the treatment of various microbial infections [43, 44]. The findings of Plenagl et al. [43] study indicated that Hyp encapsulated in fusogenic liposomes decreased the bacterial growth of *Staphylococcus saprophyticus* subsp. bovis to 2.3–2.5 log_10_ CFU/mL. In another study, using polycaprolactone-poly (ethylene glycol) NPs loaded with Hyp achieved a reduction in both planktonic and methicillin-resistant *S. aureus* (MRSA) biofilm survival by 40 and 100%, respectively [44]. According to the research, HypNP has several desirable properties such as a high quantum yield of singlet oxygen generation, preferential localization in the target sites, and a relatively low dark toxicity that minimizes its side effects, and its photo-sonosensitizing effects have been recognized previously [30–33].

Recently, D-amino acids have been proven to possess specific biological functions. Besides their therapeutic potential, some D-amino acids were shown to inhibit bacterial growth [45–47]. Caparrós et al. revealed that growth of *Escherichia coli* in the presence of D-methionine, D-tryptophan, and D-phenylalanine had no apparent effect on morphology but caused a significant inhibition of peptidoglycan synthesis and cross-linking, leading to a reduction in the growth and cell death at high concentrations [48]. In a recent study, synthesized D-proline membrane possessed good biocompatibility and could significantly promote skin wound healing, reduce the influence of bacteria on the liver and spleen in vivo, and alleviate local tissue inflammation [49]. Recent work by Ye et al. shows the bactericidal activity of an antimicrobial peptides, D-GL13K, which was derived from the human salivary against Gram-negative bacteria, e.g., *E. coli* and *Pseudomonas aeruginosa*; and Gram-positive bacteria including *Enterococcus faecalis* and *Streptococcus gordonii*. The higher bactericidal potency of AMPs containing D-amino acid attributed to their increased stability against bacterial s proteases [50]. Kan et al. [51] demonstrated that D-Trp, acting as an incompatible solute, can inhibit bacterial growth, especially for Gram-negative bacteria. In another study, Rumbo et al. [52] showed that D-Trp inhibited the growth of pathogenic bacteria as well as biofilm formation and adhesion to eukaryotic cells. ELafify et al. [53] reported D-Trp has an inhibitory effect on the growth of *Listeria monocytogenes* under different stress conditions. Also, the results of the study by Chen et al. [54] displayed that adding a small amount of D-Trp inhibits the growths of *Vibrio parahaemolyticus* and *V. vulnificus* in a high-salt environment. The antimicrobial effect of D-Trp might be involved in the inhibition of microbial growth by reducing the initial adhesion between cells and by changing the properties of the extracellular matrix, which has a considerable role in protecting the microorganisms from environmental insults [55]. Thus, HypNP represents a potent natural alternative to chemically synthesized photo-sonosensitizers.

Previous studies [56–58] verified the photoinactivation of *S. aureus* and *E. coli*, testing Hyp activated by an amber LED, similar to the present study. García et al. investigated the antimicrobial photodynamic activity of Hyp against methicillin-susceptible and resistant *S. aureus* biofilms. They showed that Hyp-photoactivity was correlated to the inactivation of staphylococcal biofilms forming [59]. Photoinactivation action of Hyp was also observed in the study by Bernal et al. [57], who reported Hyp at the concentrations of 0.1–0.4 μg/mL and LED 590 nm with 6 J/cm^2^ could kill two species of Candida, *C. albicans* and *C. dubliniensis*. A study by Montanha et al. revealed that Hyp encapsulated in Pluronic P123 copolymer micelles could inactivate *S. aureus* and *E. faecalis* following light irradiation [60]. Also, the antimicrobial results obtained by Paz-Cristobal et al. [61] and Barroso et al. [62] studies indicate that Hyp photosensitizer is an efficient choice for photodynamic inactivation against both bacteria and yeasts.

Recently, aSDT was introduced as an alternative therapy that can penetrate deeply into the target site due to its much lower attenuation coefficient in the target site compared with light sources in aPDT [63]. Several studies have shown that aSDT could be efficient in removing microbial biofilms [64–67]. According to the study by Liu et al. [64], the density of *A. baumannii* biofilms treated with aSDT using 8 μg/mL Colistin/Vancomycin was reduced by 3.77 log. Pourhajibagher et al. [65] reported that ultrasound waves enhanced the anti-caries activity of nanomicelle curcumin. In this study, aSDT treatment with 1.56 W/cm^2^ of ultrasound resulted in a drop in the viability of *S. mutans* up to 6 log. Alves et al. [66] found that the sonodynamic action of curcumin could decrease 1.7 log of *S. aureus* biofilms. Moreover, the results of a previous study [67] showed that the CFU/mL of periopathogens biofilms decreased by 6.6 log following treatment with ultrasound and chitosan nanoparticles-indocyanine green. According to the literature reviews, there are no significant studies on Hyp-mediated aSDT. Anti-cancer activity of SDT using Hyp was evaluated in many studies [68–72]; however, the effectiveness of Hyp as a potential sonosensitizer for aSDT remains relatively unknown.

Due to the increasing need for the effective treatment of *A. baumannii* infections, more attention in this study has been focused on the simultaneous use of aPDT and aSDT, which is called aPSDT against *A. baumannii*. To our knowledge, not only there was no evidence of aSDT application against *A. baumannii* in planktonic and biofilm states, but there was also no study on the synergistic effect of aPDT with aSDT methods on *A. baumannii* growth inhibition. Herein, we used HypNP@D-Trp-mediated aPSDT for investigating its antimicrobial and anti-biofilm activities against *A. baumannii*. First, we revealed a synergistic activity in the combination of HypNP and D-Trp against *A. baumannii* using a checkerboard assay. As data shown, the MIC value of HypNP and D-Trp for *A. baumannii* were ≥ 64 μg/mL and ≥ 50 mM, respectively, and the FIC index value (0.5) was within the acceptable range for the synergistic effect. The results of planktonic cultures in this study showed that aPDT and aSDT using 1/2 × MIC of HypNP@D-Trp significantly reduced the number of viable cells of *A. baumannii* by 3.20 and 3.47 log_10_, respectively. Next, we verified that aPSDT mediated by HypNP@D-Trp was able to decrease the *A. baumannii* cells in the planktonic state as compared with the control group. The cell viability of *A. baumannii* was significantly decreased by 5.10 log_10_ after treatment with aPSDT. In the current study obtained results revealed a significant biofilm degradation of *A. baumannii* after treatment with aPSDT using HypNP in combination with D-Trp. This result is consistent with recent studies of Meng et al., which demonstrate that L-lysine, L-histidine, and L-arginine porphyrin conjugates exhibit high photoinactivation efficacy against both Gram-negative and Gram-positive bacterial strains [73].

Lim et al. [74] revealed that conjugating 151-hydroxypurpurin-7-lactone dimethyl ester (G2), a semisynthetic photosensitizer, for the PDT treatment of cancer with lysine and aspartic acid amino acids moieties improved the aqueous solubility and reduced the risk of aggregation in the vascular system of G2 without affecting its photophysical characteristics. In recent work, the excessive ROS generation following near-infrared laser irradiation of doxorubicin-loaded polypeptide (poly-glutamic acid and poly-l-Lysine)-based multilayer assembled gold nanorod exhibited the ovarian cancer cells apoptosis [75]. Cheng et al. [76] evaluated a photosensitizer along with a nuclear localization sequence through an exosomal vector for PDT, which enhanced ROS effect and improved anti-tumor efficacy. It has also found that modification of ruthenium as a photosensitizer by taurine, an abundant amino acid in the brain, can be enhanced by the efficiency of PDT with boosted generation of ROS in brain cancer cells [77].

After that, we assessed the anti-biofilm activity of HypNP@D-Trp-mediated aPSDT against *A. baumannii* biofilm. Consistent with the previous studies [64–67], the findings of this study showed that HypNP@D-Trp in combination with ultrasound waves significantly degraded *A. baumannii* biofilm by 53.4% as compared to the control group.

As it has been proven, AbaI plays an important role in the biofilm formation of *A. baumannii* [78]. Targeted aPSDT may significantly reduce the pathogenicity of this bacterium following the reduction of *abaI* gene expression. Herein, the in silico analysis was conducted to analyze the molecular docking of Hyp against AbaI of *A. baumannii*. The findings of this study utilizing virtual screening methods showed that Hyp interacted with AbaI with the lowest binding energy (− 9.41 kcal/mol), which can be concluded that aPSDT using HpyNP@D-Trp can target purposefully *A. baumanii* AbaI. Our qRT-PCR results showed that the expression level of *abaI* gene in *A. baumannii* was downregulated after exposure to HypNP@D-Trp-mediated aPSDT group. Downregulation of this gene will thereby disturb the integrity of cell walls, adhesion promotion as well as biofilm formation which results in the reduction of the pathogenicity of *A. baumannii* [79]. We also showed here significant reductions of *abaI* gene in *A. baumannii* following SSD, aPDT, and aSDT treatment groups.

The low mean difference values for the log CFU concentrations, biofilm cells, and *abaI* gene mRNA level of the *A. baumannii* ATCC 19606 following each treatment, indicate that our obtained data were repeatable. Since in the current study, each assay did not have multiple independent experiments, it is not possible to discuss the reproducibility of the findings, and this is one of the limitations of the present study.

Overall, our findings suggest that the use of HypNP@D-Trp as an antimicrobial photo-sonosensitizer in the aPSDT process can be considered a prominent strategy to overcome pathogenic *A. baumannii*. Nevertheless, further in vivo studies are required to evaluate the possible potency of HypNP@D-Trp-mediated aPSDT to apply suitable alternatives for antibacterial agents in *A. baumannii* infections.

## Conclusion

In silico computational models in the current study indicated that the modeled structure of AbaI is a reliable target for HypNP in targeted HypNP@D-Trp mediated aPSDT. The value of FICI ≤0.5 revealed a synergistic effect between D-Trp and HypNP against *A. baumannii*. Under HypNP@D-Trp mediated-aPSDT, *abaI* gene mRNA level of the *A. baumannii* was down-regulated by 10.32-fold. There were significant reductions in the log CFU concentration (3.56 ± 0.7) and biofilms (53.4%) of the *A. baumannii* following treatment with HypNP@D-Trp mediated-aPSDT. Therefore, the null hypothesis was rejected. Our findings warrant a clinical trial study toward efforts to manage and also to reduce the disease severity of *A. baumannii* infections using targeted HypNP@D-Trp mediated aPSDT.

## Data Availability

All data of this study are included in the manuscript. All figures are original images and have been used for the first time in this study.

## Abbreviations

AMP: Antimicrobial peptide
aPDT: Antimicrobial photodynamic therapy
aSDT: Antimicrobial sonodynamic therapy
aPSDT: Antimicrobial photo-sonodynamic therapy
CFU: Colony forming unit
D-Trp: D- Tryptophan
HypNP: Hypericin nanoparticle
MIC: Minimum inhibitory concentration
QS: Quorum sensing
